# Determination of genomic DNA sequences for beta-tubulin isotype 1 from multiple species of cyathostomin and detection of resistance alleles in third-stage larvae from horses with naturally acquired infections

**DOI:** 10.1186/1756-3305-2-S2-S6

**Published:** 2009-09-25

**Authors:** Sarah L Lake, Jacqueline B Matthews, Ray M Kaplan, Jane E Hodgkinson

**Affiliations:** 1 Department of Veterinary Pathology, Faculty of Veterinary Science, University of Liverpool, Liverpool, L69 7ZJ, UK; 2 Parasitology Division, Moredun Research Institute, Pentlands Science Park, Bush Loan, Midlothian, EH26 0PZ, UK; 3 Department of Infectious Diseases. College of Veterinary Medicine, University of Georgia, Athens GA 30602, USA

## Abstract

**Background:**

Genetic resistance against benzimidazole (BZ) anthelmintics is widespread in cyathostomins, the commonest group of intestinal parasitic nematodes of horses. Studies of BZ-resistant nematodes of sheep, particularly *Haemonchus contortus*, have indicated that an anthelmintic resistance-conferring T/A polymorphism, encoding an F (phenylalanine) to Y (tyrosine) substitution, in beta-tubulin isotype 1 is present at two loci, codons 167 and 200 (F167Y, F200Y). Recent studies using complementary (c) DNA derived from BZ-susceptible and -resistant cyathostomins identified statistical differences in the frequency of the BZ-resistant A allele at these loci. However, the lack of high-throughput genomic DNA-based detection of polymorphisms limits the study of eggs or larvae from field isolates. In the present study, we report genomic DNA sequences for beta-tubulin isotype 1 from multiple cyathostomin species, thus facilitating the development of pyrosequencing assays to genetically characterize third-stage larvae (L3s) of cyathostomins from mixed-species field isolates.

**Results:**

Sequence analysis of the beta-tubulin isotype 1 gene in a common species, *Cylicocyclus nassatus*, indicates a revised genomic structure to published data, revealing that codons 167 and 200 are located on separate exons. A consensus sequence was generated from 91 and 76 individual cyathostomins for the regions spanning codons 167 and 200, respectively. A multi-species genomic DNA-based assay was established to directly pyrosequence individual L3 from field samples of unknown species and BZ sensitivity in a 96-well plate. In this format, the assay to detect F167Y gave a 50-90% success rate. The optimisation of the assay at codon 200 is currently underway. Subsequently, the genotype at F167Y was determined for 241 L3s, collected prior to and after BZ treatment. These results demonstrated a reduction in the heterozygous genotype, TTC/TAC, and an increase in the homozygous resistant genotype TAC/TAC in post-treatment samples. However, the differences in allele frequencies determined before and after BZ treatment were not statistically significant.

**Conclusion:**

Extensive genomic DNA sequence, spanning codons 167 and 200 of the beta-tubulin isotype 1 gene, was generated from multiple cyathostomin species. The data facilitated the development of a pyrosequencing assay, capable of detecting the genotype of individual cyathostomin L3s derived from mixed-species field samples. Differences in codon 167 allele frequencies were observed in L3s isolated pre- and post-BZ treatment.

## Background

Cyathostomins (Strongylida) are the commonest group of large intestinal nematodes infecting horses worldwide [1,2]. Parasites within this group have direct life cycles in which eggs are passed in the faeces into the environment where infective third stage larvae (L3s) develop. Upon ingestion, larvae encyst and moult to fourth stage larvae (L4s) in the large intestinal wall before emerging into the lumen, where they develop to adult stages. Large larval encysted burdens can be responsible for a life-threatening colitis, called larval cyathostominosis, with affected horses displaying clinical signs of diarrhoea, colic, inappetance and weight loss [3-5]. Approximately 50% of such cases die [6]. The epidemiology and control of cyathostomin infections is complicated by the presence of multiple species [7-9], the ability of larvae to undergo hypobiosis in the large intestinal mucosa [10] and by increasing levels of anthelmintic resistance.

Anthelmintics have been the cornerstone of cyathostomin control for the past 40 years. The benzimidazoles (BZs), followed by the tetrahydrapyrimidines and the macrocylic lactones have all been demonstrated to have high (>90%) efficacy against adult stages [11]. Frequent anthelmintic treatments, combined with their continued use over time, have resulted in considerable selection pressure for resistance and, as for ruminant parasitic nematodes, such as *Haemonchus contortus *and *Teladorsagia circumcincta*, resistance to BZs is particularly prevalent [1]. Early molecular studies of the BZ target molecule, beta-tubulin isotype 1, in *H. contortus *have revealed a single nucleotide polymorphism (SNP), an A to T transversion, resulting in a phenylalanine (F) to tyrosine (Y) substitution at codon 200 (F200Y), in BZ resistant parasites [12]. Subsequently, this mutation was shown to confer a functional resistance phenotype in *Caenorhabditis elegans *[13] and has been associated with BZ resistance in populations of *H. contortus *and *Trichostrongylus colubriformis *[14] and *Te. circumcincta *[15-17]. More recently, a similar A to T transversion at codon 167 of beta-tubulin isotype 1 has been observed in *Te. circumcincta *and cyathostomins [18,19], with the suggestion that it may be the primary mechanism of BZ resistance in the latter [20,21]. Recent studies in our laboratories have sought to detect SNPs at codons F167Y and F200Y using cDNA derived from adult cyathostomins by pyrosequencing [22]. This work supported a role for both mutations in BZ resistance in these parasites and highlighted the need to further understand their mode of inheritance [22]. Thus far, there have been few published methods to detect resistance-associated mutations in genomic DNA derived from eggs and larvae collected from the field. In the current study, we generated genomic DNA sequence data for regions spanning the 167 and 200 codons in the beta-tubulin isotype 1 gene for 13 cyathostomin species. The data obtained was then used to design a degenerate pyrosequencing assay for an analysis of the frequency of SNPs at codons 167 and 200 in mixed-species populations of third stage larvae (L3).

## Methods

### Parasites

In order to develop a robust diagnostic test to detect resistance alleles, it was necessary to generate beta-tubulin isotype 1 sequence data from multiple isolates from distinct geographical locations. To this end, sequence data for regions encoding codon 167 and 200 was generated from seven distinct isolates (1-7): (**1**) adult parasites of known FBZ resistance, (courtesy of Prof. R. M. Kaplan, University of Georgia; [23,24]) and identified to species based on morphological criteria; "A practical method of identification of the North American cyathostomes (small strongyles) in equids in Kentucky" (Tolliver S.C.); (**2**) adult parasites collected from a single horse following necropsy at the University of Liverpool (courtesy of Dr J. Cox) and identified to species using a PCR-based enzyme-linked immunosorbent assay (ELISA) [25]; (**3**) adults collected from multiple horses from an abattoir (NorthWest UK) identified morphologically to species as described in ref [25]; (**4**) L3s (designated DL3) from leisure horses on the Wirral, UK (courtesy of Professor D. Williams, University of Liverpool); (**5**) L3s (designated ILPHL3) from horses based at the International League for the Protection of Horses, Snetterton, (Norfolk, UK); (**6**) L3s (designated JCL3) from horses at Liverpool University Field station (Wirral, UK); **7**, L3 (designated WKL3) from a population of Welsh and Konik ponies (courtesy of M. Slote and S. McQueen, Norfolk Wildlife Trust/Broads Authority, UK). L3s were cultured from faecal material at room temperature for 10-14 days and collected using the Baermann technique [26]. Populations of L3s were used to increase the number of locations from which sequence data was generated. All L3s were morphologically identified as cyathostomins however the species of each individual L3 was not known. Therefore, each L3 was subject to PCR-ELISA [25] designed to detect six common species, *Cylicocyclus ashworthi*, *Cylicocyclus nassatus*, *Cylicocylcus leptostomum*, *Cylicocyclus insigne*, *Cylicostephanus longibursatus *and *Cylicostephanus goldi*, (the probe for a seventh species *Cyathostomum catinatum *was not used as the positive control values consistently did not fall above the validated cut-off). A total of six were identified as one of these species, but it was not possible to identify the remaining 34 as either they were recognised by one of the species-specific probes but did not give values above the PCR-ELISA cut-off or they were not recognised by any of the species-specific probes. As such they were known only as cyathostomin species (designated as *Cyathostominae *spp in EMBL). All parasites were stored at -20°C in absolute, molecular-grade ethanol.

### Isolation of genomic DNA from adult parasites and L3

Genomic DNA was isolated from each adult parasite using one of three methods; a modified phenol chloroform extraction method [25], Tri Reagent (Sigma-Aldrich Company, UK) or QIAmp DNA Mini Kit (Qiagen Ltd, UK). DNA pellets were re-suspended in 15 μl, 30 μl or 50 μl of 18.0M ohm water (Sigma-Aldrich Company, UK), respectively. Cyathostomin L3s were exsheathed in 0.05% sodium hypochlorite solution for 10 min and then washed twice in tap water, centrifuged for 2 min at 1000 *xg*. Individual larvae were then transferred to 0.2 ml centrifuge tubes containing lysis buffer (50 mM KCl, 10 mM Tris pH 8.3, 2.5 mM MgCl_2_, 0.45% Nonidet P-40, 0.45% Tween-20, 0.01% gelatine and 100 μg/ml Proteinase K, Sigma), frozen at -80°C for 10 min then incubated overnight at 60°C. Proteinase K was inactivated following lysis by heating to 94°C for 15 min and 25 μl of lysate were used in PCR in a volume of 50 μl.

### Sequencing of the beta-tubulin isotype 1 gene from Cylicocyclus nassatus and amplification of exons 4 and 5 from three additional species of cyathostomin

Oligonucleotide primers were designed based on the published *Cyc. nassatus *beta-tubulin isotype 1 genomic DNA sequence (Genbank:AF181093). The aim was to amplify the gene in three overlapping fragments: 5' UTR to exon 4 (5primef, 5' - AAGTTCTCTACTGCAATAATGCGTG-3' and 4r, 5' - GACCTTTGGTGAGGGAACAAC-3', annealing at 59°C); exons 4 to 6 (4f, 5'-CAGGGCTTCCAGCTAACTCACTC-3' and 6r, 5'-CCAGACATTGTTACAGAC-3', annealing at 57°C) and exon 5 to the 3' UTR (3primef, 5'-TCCGACACCGTTGTGGAG-3' and Cn31r, 5'-AACGCAATCAATGTGTATTTCGC-3' [27], annealing at 60°C). All primer combinations were used in a standard 50 μl PCR containing 1× Optibuffer, 4 mM MgCl_2_, 1× High Specificity Additive, 4 U BIO-X-ACT Long Polymerase (Bioline Ltd, UK), 0.8 mM dNTP Mix (ABgene, UK) and 1 μl of genomic template. Forty cycles were performed, following standard cycling conditions for the BIO-X-ACT polymerase at the annealing temperature defined for each primer set. PCR products were cloned into pGEM-T Easy (Promega Corporation, USA) and transformed into JM109 *E. coli *competent cells (Promega Corporation, USA) following the manufacturer's instructions. Clones were sequenced in both directions (Cogenics, USA). If necessary, internal gene-specific primers were designed to provide sequencing of full-length PCR products. Contiguous sequences were generated using the ClustalW multiple sequence alignment function in BioEdit (Hall, 1999) and Genewise  was used to define exon/intron boundaries. Initially, a full-length sequence was amplified from one *Cyc. nassatus *individual, however, following identification of a putative intron between exons 4 and 5, this region was amplified from two additional *Cyc. nassatus *individuals and each of two individuals from three additional common species, *Cyathostomum catinatum*, *Cylicostephanus goldi *and *Cylicostephanus longibursatus*. All PCR products were separated by electrophoresis in 0.8-2% w/v agarose gels (QA-Agarose Molecular Biology Grade, QBiogene, Europe) using 1× TAE buffer and containing 1× Sybr Safe DNA Gel Stain (Molecular Probes, Invitrogen). Amplicons were detected using the Syngene Gel Doc System (Syngene, Synoptics Group) using a Safe Light transilluminator (Invitrogen, UK).

### Sequencing of the region flanking codons 167 and 200 from multiple species of cyathostomins

In order to develop an assay to detect SNPs at F167Y and F200Y in populations of cyathostomins of unknown species it was necessary to avoid a bias for particular species and ensure PCR amplification and subsequent pyrosequencing of genomic DNA from multiple species of cyathostomin. It was essential to develop the assay based on sequence information for codons 167 and 200 from as many species of cyathostomin as possible. Using sequence data generated in this study, two PCR reactions were designed to amplify the regions of genomic DNA spanning codons 167 (3f, 5'-AGGATGTGACTGTCTGCAGGT-3' and 4r) and 200 (3primef and either 6r or Cn31r). Each 50 μl reaction contained 1.25 U 'Thermo-start' DNA polymerase, 1× high performance buffer, 1.5 mM MgCl_2_, 0.2 μM oligonucleotide primers, 0.8 mM dNTP mix ABgene UK (Thermo Fisher Scientific Inc.) with either 1 μl adult cyathostomin DNA or 25 μl of L3 lysate. PCR conditions were as follows: 95°C for 15 min, followed by 40 cycles of 95°C for 15 s, 30 s at 62°C and 60°C annealing for codons 167 and 200, respectively, and 72°C for 1 min, with a final extension of 72°C for 10 min. For L3 lysates and *C. goldi *adults, a second round of PCR was required, using 1 μl of the primary PCR as template in a semi-nested PCR, in which one primer was internal compared with the forward primer used in the first round of PCR. For the codon 167 region, the primers used in the first round of PCR were as stated above with primers 2f (5'-CAGGGCTTCCAGCTAACTCACTC-3') and 4r in the second round. For codon 200, primers 4f and CN31r and 4f and 6r were used in the first and second rounds, respectively. In addition, to the standard, no-template negative control reaction, the no-template control of the primary PCR was also subjected to a second round of amplification. Direct sequencing of PCR products was performed by GATC-biotech (Germany). PCR primers 3f or 2f were used for sequencing the codon 167 region, whilst the codon 200 region sequencing was performed using either primer 4f or 6r. A consensus sequence was generated from sequences determined for each species/L3 isolate, which were aligned to identify degeneracy. The aim of this study was to identify sequence degeneracy; hence, every nucleotide substitution detected, even if present in only one individual, was accepted and subsequently represented in the consensus sequence. A consensus sequence for the region encoding 167 and 200 was produced from 91 and 76 individuals, respectively. Consensus sequence, alignments and inter-specific variation for the codon 167 and 200 regions were generated using BioEdit (Hall, 1999).

### PCR and pyrosequencing of codons 167 and 200 in multiple cyathostomin species

The 167 and 200 region consensus sequences obtained were used to design primers for PCR and pyrosequencing. A semi-nested PCR was used for the codon 167 region involving a degenerate version of the 3f primer, located at the end of exon 3, 167f (5'-AGGWTGYGAYTGTCTKCARG-3') and a degenerate version of primer 4r, 167r (5'-RMCCTTTGGTGARGGRACAAC-3') in the first round, followed by primer 167r with a degenerate version of 4f, 167fs (5'-CAGGGYTTCCARYTAACTCACTC-3') in the second round. The codon 200 region was amplified using primers, 200f (5'-TCYGAYACHGTTGTGGAGCC-3') and a degenerate version of primer 6r, 200r (5'-ACSCCAGACATTGTTACAGAC-3'). Five μl of genomic DNA template were subjected to 40 cycles of PCR (as described above), with an annealing temperature of 62°C and 60°C for codons 167 and 200, respectively. For both codons, the reverse primers were biotinylated (MWG Biotech AG) and 10 μl of PCR product were resolved by agarose gel electrophoresis. PCR products were subjected to pyrosequencing with primers 167seq (5'-ATAGAATYATGTCYTCDW-3') and 200seq (5'-TTGAARATACAGRCKWRACT-3'). Reactions were performed on a PSQ™ 96MA instrument (Biotage™) according to the manufacturer's instructions. Forty μl of biotinylated PCR product and 1 μM of sequencing primer were used in each reaction.

The no-template (negative) control for each PCR and positive controls (known genotypes) were run on each plate. To ensure the reproducibility of the PCR and pyro-sequencing, products from five adult parasite DNA samples were repeated in both assays. Identical results were obtained for all samples tested (data not shown). The steps for the amplification and pyrosequencing of individual L3s (collected from faecal culture and lysed, as described previously) were essentially the same as for adults, except that 25 μl of lysate were used in each PCR and, for codon 200, a semi-nested PCR was employed using primers pair 200f-200r and primer pair 200f-200rs (5'-AAGATGATTYAVATCWCCRTA-3') in the first and second rounds, respectively. In both rounds of the codon 200 PCR, 1 μM of each primer was used, employing an annealing temperature of 60°C. Initially, a subset of L3s was PCR amplified and pyrosequenced for either codon prior to higher throughput analysis of L3s in a 96-well plate format for codon 167.

### Determination of genotypes at codon 167 in two isolates of cyathostomin L3, pre- and post-anthelmintic treatment

Cyathostomin L3s exposed previously to fenbendazole (FBZ), ivermectin, oxibendazole and pyrantel were cultured and harvested during a large-scale survey of the prevalence of anthelmintic resistance in the USA [28]. The pyrosequencing assay developed here was used to determine the genotype at codon 167 for a total of 241 L3 from four isolates within this archive, including L3s obtained pre- and post-treatment with FBZ from one location (1) in Louisiana and one location in Florida (2), USA, both of which displayed resistance to FBZ based on the results from faecal egg count reduction testing [28]. The L3s were lysed, PCR performed directly on lysate and their genotype was determined by pyrosequencing for F167Y in a 96-well plate format. Typically, genotypes were able to be determined for 50-90% of the L3 on each plate. All control reactions were as described above.

### Statistical analysis

Differences in genotype and allele frequencies among populations were analysed statistically using the Mann-Whitney and Fisher's exact tests, employing SPSS software (v 13.0.1., 2004). In all cases, a critical probability (*P*) of < 0.05 was considered statistically significant.

## Results

### Organisation of the beta-tubulin isotype 1 gene of *Cylicocyclus nassatus*

**Figure 1:**
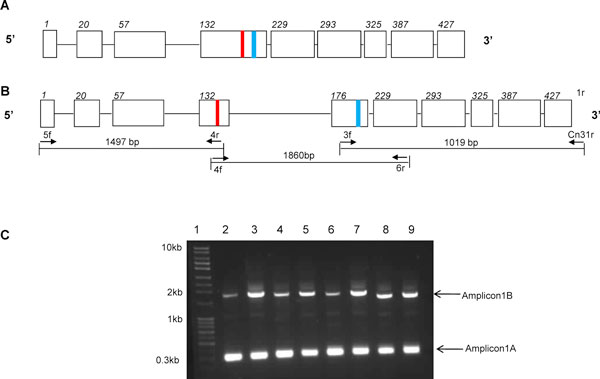
**Genomic organisation of *Cylicocyclus nassatus *beta tubulin isotype 1**. **(a) **Schematic of published gene [29] showing order and size of exons and introns. **(b) **Schematic of proposed gene, showing alternative organisation of exons and introns and position of primers used to amplify three overlapping fragments used to generate contiguous sequence. Exons and introns are represented by boxes and lines, respectively and number of the first amino acid residue of each exon is shown in italics. The position of codons 167 and 200 are marked red and blue boxes, respectively. Schematics are not to scale. **(c) **PCR using primers 4f and 6r, spanning exons 4 and 5, from genomic DNA template showing co-amplification of two alternative amplicons from four species of cyathostomin. Lanes: 1, Quantitative ladder 4 (Yorkshire Bio, UK), 2 and 3 *Cylicocylus nassatus*, 4 and 5 *Cyathostomum catinatum*, 6 and 7 *Cylicostephanus longibursatus*, 8 and 9 *Cylicostephanus goldi*. Two products were amplified for each parasite at ~300 bp and ~1800 bp.

According to the previously published sequence of the beta-tubulin isotype 1 gene of *Cyc. nassatus *[29], the regions encoding codons 167 and 200 lie within exon 4 (Figure 1a). Based on this information, a PCR assay was designed to amplify this region using genomic DNA extracted from each of four common cyathostomin species, *Cyc. nassatus, Cys goldi*, *Cya catinatum *and *Cys longibursatus*. The PCR was either unsuccessful or two amplicons were generated (data not shown). This prompted further investigation of the isotype 1 gene of *Cyc. nassatus*. As shown (Figure 1b), the gene was amplified in three overlapping regions; a 1860 bp amplicon generated with primers 4f and 6r, a 1497 bp product generated using primers 5f and 4r at the 5', and at the 3' end, a 1010 bp product generated with primers 3f and Cn 31r. The three amplicons were sequenced and aligned to give a consensus sequence of 4146 bp. Alignment of the gene with a cDNA sequence from the homologous species [accession number EU251453] revealed that exon/intron boundaries were identical to those predicted for the previously published *Cyc. nassatus *sequence [29], with the exception of an additional intron at nucleotide position 1497 (amino acid codon 175). As a result of this analysis, we propose a modified genomic organisation for *Cyc. nassatus *beta-tubulin isotype 1, as depicted schematically in Figure 1b. Subsequently, the primer pair 4f and 6r (flanking exons 4 and 6) were applied to several species and consistently produced two amplicons of ~300 bp and ~1800 bp (Figure 1c). These two products were not cloned and sequenced. However, the 300 bp product was consistent in size with the region spanning exons 4, 5 and 6 from the published gene [29] (Figure 1a), whilst the ~1800 bp product is the same as that of the alternative gene predicted here (Figure 1b), suggesting that two arrangements of this gene exist in cyathostomin species.

### Sequencing of the beta-tubulin isotype 1 region encoding codons 167 and 200 from multiple species

**Figure 2:**
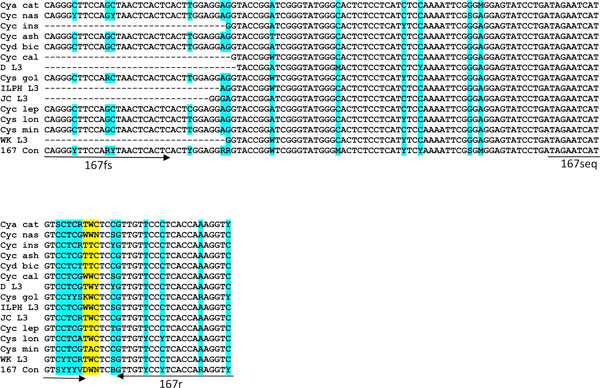
**Multiple species/isolate alignment of nucleotide sequence for exon 4 encoding codons 132-175**. Primers for a multi-species pyrosequencing assay are indicated by arrows in the forward and reverse direction (167fs and 167r) and for the pyrosequencing primer (167seq). The first round forward PCR primer (167f) is not shown. The codon for F167Y is indicated in yellow. Sequence degeneracy is indicated in blue by the relevant nucleotide code: R = A/G, Y = C/T, K = G/T, M = A/C, S = C/G, W = A/T, B = C/G/T, D = A/G/T, H = A/C/T, V = A/C/G, N = any. Consensus sequences were generated from the following species; *Cyathostomum catinatum *(Cya cat), *Cyclicocyclus nassatus *(Cyc nas), *Cylicocylus insigne *(Cyc ins), *Cylicocylcus ashworthi *(Cyc ash), *Cylicodontophorus bicoronatus *(Cyd bic), *Cylicocylcus calicatus *(Cyc cal), *Cylicostephanus goldi *(Cys gol), *Cylicocyclus leptostomum *(Cyc lep), *Cylicostephanus longibursatus *(Cys lon), *Cylicostephanus minutus *(Cyc min) and for four L3 populations of unknown species, DL3, ILPHL3, JCL3 and WKL3. The 167 consensus (167 con) sequence is shown at the bottom of the alignment.

**Figure 3:**
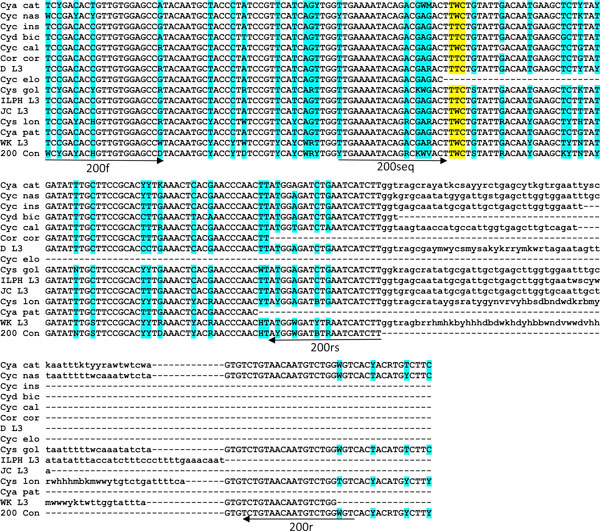
**Multiple species/isolate alignment of nucleotide sequence for exons 5 and 6, encoding codons 176-240 and intron 5**. Primers for a multi-species pyrosequencing assay are indicated by arrows in the forward and reverse direction (200s and 200r) and for the pyrosequencing primer (200seq). The second round reverse PCR primer (200rs) used for L3 is shown. Coding region of exons 5 and 6 are shown in upper case with F200Y indicated in yellow and intron sequence is shown in lower case. Sequence degeneracy is indicated as shown for Figure 2. Consensus sequences were generated from the species indicated in Figure 2 with the absence of *Cylicocyclus leptostomum *(Cyc lep), *Cylicostephanus minutus *(Cyc min), *Cylicocylcus ashworthi *(Cyc ash) and the addition of the following three species; *Coronocyclus coronatus *(Cor cor), *Cylicocyclus elongatus *(Cyc elo), *Cyathostomum pateratum *(Cya pat). The 200 consensus (200 con) sequence is shown at the bottom of the alignment.

To develop a pyrosequencing assay capable of detecting F167Y and F200Y in "field samples" containing mixed species, it was essential to design primers based on the sequences from multiple species and isolates. This data was generated using a combination of genomic DNA and cDNA sequence data for multiple individual adults and L3s. For the region encoding codon 167, the following parasites were used, 49 sequences from adult parasite genomic DNA, 21 sequences from genomic DNA from L3s and 21 sequences of cDNA published previously [22]. A consensus sequence was produced for the following species; *Cyathostomum catinatum *(n = 17), *Cyclicocyclus nassatus *(Cyc n = 14), *Cylicocylus insigne *(n = 9), *Cylicocylcus ashworthi *(n = 1), *Cylicodontophorus bicoronatus *(n = 1), *Cylicocylcus calicatus *(n = 4), *Cylicostephanus goldi *(n = 17), *Cylicocyclus leptostomum *(n = 1), *Cylicostephanus longibursatus *(n = 8), *Cylicostephanus minutus *(n = 1) and for 18 L3s not identified to species and potentially of one or more unknown cyathostomin spp., DL3 (n = 6), JCL3 (n = 3), ILPHL3 (n = 5) and WKL3 (n = 4) [accession numbers EMBL;AM930573-AM930601, AM930603-AM930643]. For the region encoding codon 200, 36 sequences from genomic DNA from adults, 19 sequences from L3s and 21 sequences from cDNA published previously [22] were used. A consensus sequence was generated from the following number of individuals for each species; *Cyathostomum catinatum *(n = 8), *Cyclicocyclus nassatus *(n = 16), *Cylicocylus insigne *(n = 1), *Cylicodontophorus bicoronatus *(n = 1), *Coronocyclus coronatus *(n = 1), *Cylicocylcus calicatus *(n = 3), *Cylicocyclus elongatus *(n = 1), *Cylicostephanus goldi *(n = 19), *Cylicostephanus longibursatus *(n = 10), *Cyathostomum pateratum *(n = 1) and for 16 L3s not identified to species and potentially of one or more unknown cyathostomin spp., DL3 (n = 3), JCL3 (n = 2), ILPHL3 (n = 5) and WKL3 (n = 6) [accession numbers; EMBL: AM930574, AM930581, AM930583AM930586, AM930588-AM930594, AM930600, AM930607-AM930608, AM930629, AM930643, AM930646-AM930656, AM930659-AM930665, AM930669, AM930673, AM930677, AM930680, AM930682-AM930689, AM930697, AM930703-AM930708]. The resultant consensus sequences were aligned for the regions of interest for each codon (Figures 2 and 3). Interspecific variation across the 97-nucleotide region for 167 (codons 143-174) ranged from 0-11% with 0-15% variation across a 137-nucleotide region for codon 200 (codons 176-221). The greatest difference was observed between *Cys goldi *and the L3 isolate DL3 and *Cys goldi *and the L3 isolate WKL3, for the 167 and 200 regions, respectively. Intra-specific variation for the coding sequence in the region of 167 and 200 was similar to previous reports [22]; however, when intron sequence was obtained for the different species, there were differences in size (see for example intron 5; Figure 3).

### Pyrosequencing assays to determine the genotype at codons 167 and 200 of beta-tubulin isotype 1 for multiple cyathostomin species

**Figure 4:**
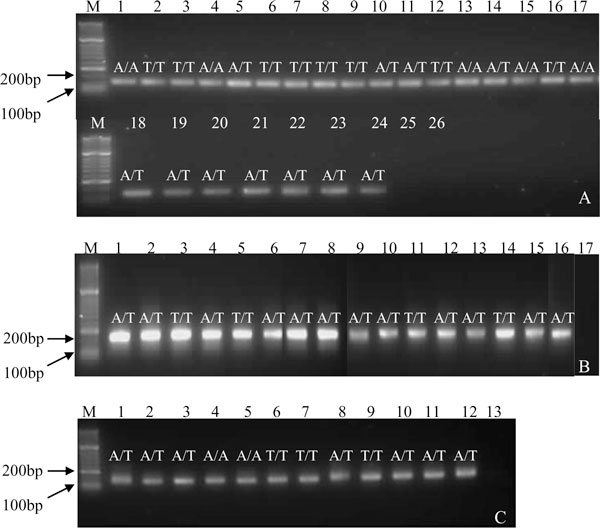
**PCR and pyrosequencing of codons 167 and 200 of beta-tubulin isotype 1 from multiple species of cyathostomin**. **(a) **PCR and genotype of F167Y for 13 species of cyathostomin. Lanes: M molecular weight marker Hyperladder II (Bioline), 1 and 2 *Cylicocyclus nassatus*, 3 and 4 *Cyathostomum catinatum*, 5 and 6 *Cylicocyclus ashworthi*, 7 and 8 *Cylicostephanus goldi*, 9 and 10 *Cylicocyclus elongates*, 11 and 12 *Cylicocylus insigne*, 13 *Cylicostephanus longibursatus*, 14 and 15 *Cylicocyclus leptostomum*, 16 and 17 *Cyathostomum pateratum*, 18 *Cylicostephanus minutus*, 19 and 20 *Cylicodontophorus bicoronatus*, 21 and 22 *Coronocyclus coronatum*, 23 and 24 *Cylicocylcus calicatus*, 25 no template control from primary round PCR, 26 second round PCR no template negative control. **(b) **Composite figure of PCR and genotype of F200Y for 10 species of cyathostomin. Lanes: M molecular weight marker Hyperladder II (Bioline), 1 and 2 *Cyc nassatus*, 3 and 4 *Cys goldi*, 5 and 6 *Cya catinatum*, 7 *Cys longibursatus*, 8 and 9 *Cor coronatum*, 10 *Cys calicatus*, 11 and 12 *Cyc ashworthi*, 13 *Cylicodontophorus bicoronatus*. 14 and 15 *Cyc leptostomum*, 16 *Cys minutus*, 17 no template negative control. **(c) **Semi-nested PCR of F167Y region for cyathostomin L3. Lanes: M molecular weight marker Hyperladder II (Bioline), 1-12 individual L3, 13 no template control. Genotypes are represented as follows:T/T, homozygous 'susceptible';A/T, heterozygous, A/A, homozygous 'resistant'.

The consensus sequence derived from multiple species was used to design a degenerate PCR as well as sequencing primers for pyrosequencing (Figures 2 and 3). To confirm potential of the assay for the multiple species, PCR products representing the regions encoding codon 167 and 200 were amplified efficiently and genotypes were determined for adult genomic DNA from two individuals of each of 13 common species for codon 167 (Figure 4a) and of 10 species for codon 200 (Figure 4b). This finding validated the degenerate nature of the primers and demonstrated that the assays provide a robust technique for determining the genotype at these loci. A single round PCR for the codon 200 region did not yield amplicons for *Cyc. insigne*, *Cya. pateratum *and *Cyc. elongatus*. Although sequence in this region was obtained for all three species, this did not cover all PCR and pyrosequencing primer sites, indicating there may be a lack of sequence conservation in this region in these species. This finding is particularly relevant as only one individual was sequenced for each of the three species (Figure 3). The codon 200 assay requires further validation on a larger cohort of parasites of known species. To demonstrate the potential of the assays for the analysis of field samples of unknown species, a subset of L3s from our archive were subject to *in vitro *lysis, PCR and pyrosequencing to determine their genotype. This analysis indicated that the PCR was sufficiently sensitive to amplify the 167 region from 12 individual L3s of unknown species identity without prior DNA extraction. Furthermore, pyrosequencing was successfully applied to all 12 L3s to reveal one of two alternative genotypes (Figure 4c). Due to the further validation of the codon 200 region, only the assay to detect F167Y was used to determine the genotype of additional L3s; however, at this stage, it was confirmed that PCR amplicons and pyrosequencing was successful for a subset of individual L3s using the codon 200 PCR protocol (data not shown).

### Determination of the genotype of cyathostomin L3s from field samples

**Figure 5:**
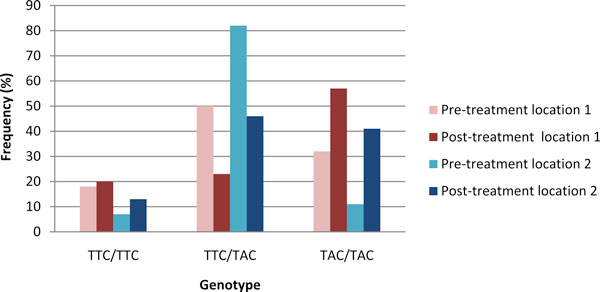
**Genotypes at F167Y for cyathostomin L3s from two locations pre- and post-benzimidazole treatment**. Genotypes were calculated as a % of the total number of parasites for which genotypes were determined at each location. Pre- and post-treatment data is shown for the two locations; location 1,"pre-treatment 1" n = 71,"post-treatment 1" n = 54, location 2,"pre-treatment 2" n = 72,"post-treatment 2" n = 44. Genotypes are represented as follows: TTC/TTC homozygous 'susceptible' encoding an F (phenylalanine) amino acid (aa); TTC/TAC heterozygous and TAC/TAC homozygous 'resistant' encoding a Y (tyrosine) aa.

Cyathostomin L3 isolates obtained before and after treatment with BZ from two farms in Louisiana, USA, were subjected to PCR and pyrosequencing for F167Y. The genotypes obtained for each isolate at locations '1' and '2' are shown in Figure 5. The results indicated that similar levels of the homozygous susceptible genotype, TTC/TTC, were present before and after treatment. Following the administration of FBZ, the results show that there was a reduction in the heterozygous genotype, TTC/TAC, with an increase in the homozygous resistant genotype, TAC/TAC, in both locations. Before and after treatment, the frequency of the resistant allele A in samples was recorded as 52% and 64%, respectively, for location '1', and 57% and 68%, respectively, for location '2'. The allele frequencies between "before" and "after" BZ treatment were not statistically different at either location.

## Discussion

Previous studies of the molecular basis of BZ-resistance in cyathostomins have addressed mutations at F167Y and F200Y using the cDNA derived from adult parasites [22,30,31], which are limited in their application, as they are usually recovered at necropsy, or from small numbers of L3s from a single isolate [32,33]. To understand the role of these mutations, it is essential to genotype large numbers of parasites of all stages, dictating the need for a genomic DNA-based assay. The present study reports genomic DNA sequence data for the beta-tubulin isotype 1 gene from multiple species and isolates of cyathostomins, allowing the design of multi-species assays that can target all stages of the parasite life cycle. Historically, the egg hatch assay (EHA) and larval development assay (LDA) have been used to examine the exposure of cyathostomin populations to differing levels of BZ drugs *in vitro *[34-37]. The combination of an *in vitro *approach with the genetic assay developed here allows the analysis of changes in allele frequency in large populations of L3s (and potentially eggs) under a direct selection pressure with the drug, which is an important study to perform in future.

The present study applied a pyrosequencing assay, used previously to determine the genotype of adult cyathostomins using cDNA [22], to genomic DNA extracted from L3s. The success of this approach relied on the F167Y and F200Y codons being in close proximity in the same exon of the beta-tubulin isotype 1 gene, as had been supported by published data [38]. Our data indicate that there are at least two copies of this gene in the cyathostomin genome; one comprising 10 exons and nine introns, similar to that of the orthologous gene in *H. contortus *[15], and a second copy consisting of a coding region with no introns (based on sequence analysis of exons 3-4 and 5-10 from *Cyc. nassatus *and *Cyc calicatus*, data not shown). We propose that the latter is a nonfunctional, processed pseudogene [39]. This information is meaningful in ensuring that the assay targets the appropriate part of the genome. For what we propose is the functional gene, the current studies indicate that these two codons appear to lie on two separate exons, separated by an ~1400 bp intron, making a single PCR-based assay to detect F167Y and F200Y untenable. The data generated here will prove central to the design of two independent assays, which can be used on the same individual parasite, thus facilitating the detection of genotypes of individuals or groups of cyathostomin of all stages.

Individual adult parasites of known species and L3s from the field (from 6 and 1 locations in the UK and the USA, respectively) were used to identify sequence variation in the region of interest. Consensus sequences were of different lengths, making comparison based sequence identity difficult across the full region; hence, a sequence directly around each codon was subsequently explored. For both codons, the greatest degree of sequence variation was observed for *Cys goldi *by comparison with one of the L3 isolates; this is probably because the *Cys goldi *consensus sequence was generated from the largest cohort of parasites (n = 17-19) and the L3 populations were collected from the field. These observations support the need to generate extensive sequence data, before any assay is designed to investigate field populations. Subsequently, the potential of an assay to detect F167Y was realised by a series of experiments using multiple species (adult stages) and samples from an L3 population whose species composition was unknown but was considered to be representative of a "typical" field sample.

We previously determined the genotype for > 170 individual adult parasites at codons 167 and 200 using cDNA derived from four cyathostomin populations, one BZ-sensitive and three BZ-resistant, and demonstrated a statistical difference in F167Y and F200Y in susceptible *versus *resistant parasites [22]. We emphasized the need to study further such populations to understand the relative role of each mutation and raised questions about the heritability of BZ resistance, not least whether these mutations are the primary mechanism of BZ resistance in cyathostomins [22]. In the present study, the detection of genotypes at F167Y for 241 L3s, involving a direct lysis and pyrosequencing in a 96-well plate format, illustrated how such an assay can be applied to field samples. The results suggest that there is a trend towards a decrease in the heterozygous TTC/TAC genotype post-BZ treatment, with a coincident increase in the TAC/TAC genotype, supporting a role for this SNP in resistance. However when analysed in terms of the overall frequency of the resistant A allele, there was no statistical difference between "before" and "after" treatment samples at both locations. Clearly, when subjected to analysis in terms of alleles rather than genotype, the resistant allele in heterozygotes contributes as well as those of the homozygous resistant genotype. The genetics of BZ resistance in the cyathostomins is not known. Although the assumption is made that it is a dominant trait, only by a series of specific experiments will it become apparent. An assay to analyse large numbers of individual L3 from multiple isolates will facilitate these studies. To this end, we are currently optimising the simultaneous detection of both F167Y and F200Y from more individual L3s, combined with the detection of both SNPs in pooled L3 samples. We aim to apply this approach to our extensive archive of L3 derived from different populations from horses with naturally acquired cyathostomin infections.

## Conclusion

The ability of the pyrosequencing assays developed here to determine the genotype of cyathostomin L3 for mutations at codon 167 and potentially codon 200 of the beta-tubulin isotype 1 gene facilitates the study of large population sizes under the direct selection pressure of BZs, either *in vivo *or *in vitro*. The data reported here support the delivery of a molecular diagnostic assay for BZ resistance in field samples and the availability of a tool to address key questions in the biology of BZ resistance in these important equine nematodes.

## Competing interests

The authors declare that they have no competing interests.

## Authors' contributions

SLL generated the sequencing data, developed the pyrosequencing assay and drafted the manuscript. JBM made significant contribution to the concept of study, experimental design and the manuscript. RMK contributed to concept of the study and experimental design particularly with respect to selection of parasite material. JEH conceived of, designed and coordinated the study and was responsible for the final manuscript. All authors read and contributed to the final manuscript.
